# The Impact of National Institutes of Health Funding on U.S. Cardiovascular Disease Research

**DOI:** 10.1371/journal.pone.0006425

**Published:** 2009-07-29

**Authors:** Radmila Lyubarova, Brandon K. Itagaki, Michael W. Itagaki

**Affiliations:** 1 Division of Cardiology, Department of Medicine, Albany Medical College, Albany, New York, United States of America; 2 Department of Cardiology, Kaiser Permanente Medical Center, Los Angeles, California, United States of America; 3 Division of Geriatric Medicine, Department of Medicine, David Geffen School of Medicine at the University of California Los Angeles (UCLA), Los Angeles, California, United States of America; 4 Department of Radiology, David Geffen School of Medicine at the University of California Los Angeles (UCLA), Los Angeles, California, United States of America; London School of Hygiene and Tropical Medicine, Peru

## Abstract

**Background:**

Intense interest surrounds the recent expansion of US National Institutes of Health (NIH) budgets as part of economic stimulus legislation. However, the relationship between NIH funding and cardiovascular disease research is poorly understood, making the likely impact of this policy change unclear.

**Methods:**

The National Library of Medicine's PubMed database was searched for articles published from 1996 to 2006, originating from U.S. institutions, and containing the phrases “cardiolog,” “cardiovascular,” or “cardiac,” in the first author's department. Research methodology, journal of publication, journal impact factor, and receipt of NIH funding were recorded. Differences in means and trends were tested with t-tests and linear regression, respectively, with *P*≤0.05 for significance.

**Results:**

Of 117,643 world cardiovascular articles, 36,684 (31.2%) originated from the U.S., of which 10,293 (28.1%) received NIH funding. The NIH funded 40.1% of U.S. basic science articles, 20.3% of overall clinical trials, 18.1% of randomized-controlled, and 12.2% of multicenter clinical trials. NIH-funded and total articles grew significantly (65 articles/year, P<0.001 and 218 articles/year, P<0.001, respectively). The proportion of articles receiving NIH funding was stable, but grew significantly for basic science and clinical trials (0.87%/year, P<0.001 and 0.67%/year, P = 0.029, respectively). NIH-funded articles had greater journal impact factors than non NIH-funded articles (5.76 vs. 3.71, P<0.001).

**Conclusions:**

NIH influence on U.S. cardiovascular research expanded in the past decade, during the period of NIH budget doubling. A substantial fraction of research is now directly funded and thus likely sensitive to budget fluctuations, particularly in basic science research. NIH funding predicts greater journal impact.

## Introduction

In a speech given at the 2006 American Heart Association national meeting, Dr. Elias A. Zerhouni, Director of the National Institutes of Health (NIH), emphasized the tremendous benefit derived from prior government funding of clinical research. Dr. Zerhouni used coronary artery disease as an example, where research has prevented one million early deaths at a cost of supporting the NIH of $3.70 per American per year. Despite these proven benefits, the likelihood of an investigator obtaining NIH research funding dropped by a third from 2003 to 2006 [1]. From 2003 to 2008, NIH budgets stagnated, and even declined in terms of real purchasing power [2], [3]. Dr. Zerhouni described the situation as a “perfect storm,” and was “deeply troubled” about the impact on investigators, particularly junior scientists beginning a career [4]. In February 2009, the issue of NIH funding again took center stage with the passage of the American Recovery and Reinvestment Act (ARRA), which promised an additional $10.4 billion to be spent by fiscal year 2010, a substantial boost for the $30.3 billion 2009 NIH budget [3], [5].

Adequate funding is critical for continued advancement in cardiovascular disease research. In 2008, the NIH budget was approximately $29 billion, with about $2.9 billion directed toward the National Heart, Lung, and Blood Institute (NHLBI), the institute most directly involved with cardiovascular research [6]. Despite these well-publicized budgetary figures, the actual impact of the NIH on published cardiovascular research is poorly understood. Money flows from the government to research institutions, but the return on that investment, in terms of the quality and quantity of research publications, is almost entirely unknown.

Quantifying the output of U.S. cardiovascular research, both NIH-funded and unfunded, is important. Doing so provides a baseline understanding of the volume and characteristics of cardiovascular research, and also allows trend analysis to determine if research productivity is increasing, decreasing, or stagnant. The relative proportion of NIH-funded to total research is also of importance. A large proportion of NIH-funded papers suggests a heavy dependence on direct NIH support. In this instance, research output may change dramatically in response to changes in grant appropriations. A small proportion suggests that research productivity is less influenced by the NIH. In the current context of a surge in available research funding secondary to the ARRA, this information has important policy implications and could impact decisions on grant appropriations. For overall U.S. biomedical research, the NIH accounts for 24% to 28% of total research funding [7]. Specialty-specific studies examining the role of the NIH in research have been performed in radiology [8], [9], emergency medicine [10], [11], otolaryngology [12], and neurology [13]. For cardiovascular disease however, the publication impact of NIH funding is unknown.

The purpose of this study was to determine the overall volume of world and U.S. cardiovascular research articles, and the component of U.S. cardiovascular articles that did and did not receive NIH funding during the eleven years from 1996 to 2006. NIH funding levels were examined overall and by research methodology to determine which methodologies were most likely to receive funding. Trends were analyzed to determine if funding impact changed over time. The average journal impact factor was examined to determine if article methodology or receipt of NIH funding was associated with the likely scientific impact of the article. Finally, the contributions of individual NIH institutes were examined to determine which ones had budgets and agendas that were most supportive of cardiovascular research. These results provide an overview of the NIH funding landscape in cardiovascular disease research and provide important information for policymakers, research leaders, and clinical investigators who are involved with research grants, and for practicing clinicians interested in continued benefits provided by research.

## Methods

### Source of Data

This study is a retrospective observational study of publicly available data, and was exempted from Institutional Review Board approval. Data collection was performed by MWI, and analysis and interpretation was performed by MWI, RL, and BKI. The data were obtained from PubMed, a freely available database of biomedical citations and abstracts hosted by the National Library of Medicine. The largest component of PubMed is MEDLINE, a database that contains information from more than 5,200 biomedical journals published in more than 80 countries, and in 37 languages [14], [15]. PubMed contains over 18.8 million citations [16].

### Data selection

All articles published during the 11-year period from January 1, 1996 to December 31, 2006 that were indexed in PubMed as of June 20, 2007 and contained the words “cardiac,” “cardiovascular,” and “cardiolog” in the first author affiliation field were captured. The author affiliation field is a PubMed defined field that contains the first author's department, institution, and country. This method allowed capture of citations published by departments of cardiology and related specialties, including surgical, pediatric, and basic science disciplines. In instances where an author was not from a cardiovascular related department but worked within a larger institution dedicated to cardiovascular research, the paper was included. Papers from veterinary medicine departments were specifically excluded as they typically deal with research unrelated to humans. The selection was thus conservative in nature, tending to exclude some legitimate articles where the first author is from an unrelated department in order to ensure that the included articles had the highest chance of being truly cardiovascular in nature. The goal of the chosen keyword selection algorithm was thus not to capture all cardiovascular articles, but rather to capture a large subset with maximum accuracy.

The matching citation data were reconstructed in a dedicated research database. For each article, the original PubMed-defined fields were preserved, including the author affiliation, year, volume, issue, journal of publication, article methodology, and NIH supporting institute, if any. Each article had one or more methodology types as defined by the National Library of Medicine: case reports, which describe clinical presentations; review articles, which examine published material on a subject; multicenter studies, which describe a controlled study performed at multiple institutions; therapeutic comparison studies, which compare therapeutic techniques and approaches; process evaluations, which examine the utility and effectiveness of processes; process validations, which study the reliability of processes; In vitro studies, which examine excised tissue; clinical trials (of any type), which describe pre-planned clinical studies typically involving humans, or controlled, randomized-controlled, Phase I, II, III, or IV clinical trials. Precise definitions of these publication methodologies are available from the National Library of Medicine [17]. Two additional methodological types were created: Research articles with no specified subtype comprise a large fraction of the published literature and typically consist of basic science laboratory or early clinical research. These articles were labeled “unspecified, general research.” Multicenter studies that were also clinical trials were given the label “multicenter clinical trial.” If the article acknowledged receipt of NIH grant support, the sponsoring NIH institute was noted. While PubMed has standardized indexing of NIH grants, no standardized method of indexing non-NIH funding exists. Thus, only NIH grants were captured. In cases where articles had support from two or more NIH agencies, each agency was given equal credit for the publication. Country assignment was based on automated detection of the country, city, or institution in the first author's affiliation field.

Journal impact factor (JIF) values of the publishing journal were obtained from Thompson Scientific Journal Citation Reports and assigned to each article by the year and journal of publication [18]. A journal's impact factor is calculated as the total number of citations in a year referencing articles from a journal's prior two years, divided by the number of articles from a journal's prior two years [19]. While not a perfect measure of an article's scientific significance, JIF has been found to be associated with article methodological strength and subsequent scientific impact [20], [21]. JIF values were assigned to individual articles using journal titles and ISSN numbers. Data were available for 7467 unique journals from 1997 to 2006. Mean JIF values for NIH-funded, non-NIH-funded, and total U.S. articles were calculated. Calculations were also performed for each article methodology. Articles without assignable JIF values were excluded from the calculations.

To quantify the accuracy of the selection criteria, randomly generated 500 citation data sets were compared to human review as a gold standard. Manual review was performed by RL. Detected errors were confirmed by consensus of the authors. In a set of articles identified as U.S. cardiovascular, 497 of 500 (99.4%) were properly classified as originating from cardiovascular or related departments or institutions, and 497 of 500 (99.4%) were properly identified as originating from the United States. In a set of worldwide articles, 500 of 500 (100%) were properly classified as originating from cardiovascular or related departments or institutions, and 479 of 500 (95.8%) had been assigned to the correct country of origin. Inability to assign country occurred primarily when incomplete information regarding the author's institution was provided.

### Data analysis

Statistical analysis was performed using SPSS software (Windows version 11.5, SPSS, Inc. Chicago, IL, USA). Ordinary least squares regression analysis was used to test for trend, that is, a nonzero slope over the 11-year study period. Two tailed t-tests were used to test for differences in mean journal impact factor values. A *P*-value of less than or equal to 0.05 was used as a threshold for significance.

## Results

A total of 117,643 cardiovascular disease journal articles published between the years 1996 to 2006 were identified from 2,982 unique journals. Of those, 36,684 articles from 1,788 journals originated from the United States and comprised the final data set. U.S. institutions published approximately one-third (31.2%) of the world total. U.S contributions met or exceeded 35% of the world total for meta-analyses, in vitro studies, process validations, multicenter studies, multicenter trials, and phase II trials. U.S. contributions exceeded 40% for review articles, phase I, and phase IV trials, and exceeded 50% for phase III trials. Less than one-quarter of the world's case reports, controlled, randomized-controlled, and overall clinical trials originated from the U.S., Table 1.

**Table 1:** Worldwide, U.S., and U.S. NIH-funded cardiovascular research articles, 1996–2006, by article study characteristics and methodology.

Study characteristics and methodology	Worldwide	U.S. (% of world)		U.S. NIH-funded (% of U.S.)	
Case reports	18,004	3,879	(21.5%)	65	(1.7%)
Review articles	18,570	7,844	(42.2%)	1,152	(14.7%)
Therapeutic comparisons	17,140	4,799	(28.0%)	1,168	(24.3%)
Process evaluations	2,685	738	(27.5%)	151	(20.5%)
Process validations	560	198	(35.4%)	57	(28.8%)
In vitro studies	2,604	937	(36.0%)	589	(62.9%)
Multicenter studies	2,682	948	(35.3%)	167	(17.6%)
Meta-analyses	370	135	(36.5%)	13	(9.6%)
Twin studies	26	0	(0.0%)	0	(0.0%)
Clinical trials, all types	10,508	2,396	(22.8%)	487	(20.3%)
Controlled clinical trials	1,216	235	(19.3%)	62	(26.4%)
Randomized controlled trials	5,889	1,403	(23.8%)	254	(18.1%)
Multicenter trials	1,622	622	(38.3%)	76	(12.2%)
Phase I trials	64	28	(43.8%)	6	(21.4%)
Phase II trials	103	36	(35.0%)	4	(11.1%)
Phase III trials	41	22	(53.7%)	0	(0.0%)
Phase IV trials	11	5	(45.5%)	0	(0.0%)
Unspecified, general research	53,157	17,078	(32.1%)	6,850	(40.1%)
Miscellaneous articles	1,233	656	(53.2%)	27	(4.1%)
All journal articles	117,643	36,684	(31.2%)	10,293	(28.1%)

Articles may belong to more than one category, thus the sum of the categories do not necessarily equal the total. *Unspecified, general research* are research articles with no specified subtype. *Miscellaneous articles* are journal articles without scientific content, such as bibliographies, biographies, comments, letters, historical articles, guidelines, editorials, news, indices, legal cases, interviews, and consensus statements. Additional information on publication characteristics and methodologies is available at the National Library of Medicine website, at http://www.nlm.nih.gov/mesh/pubtypes2008.html.

Significant growth was seen for overall U.S. cardiovascular publications over the 11-year study period, with publications increasing an average 218 articles per year (*P*<0.001). Significant growth was seen for all methodological subtypes except in vitro studies and phase-type trials, and was particularly great for review articles (72.8 articles/year), Table 2. Process evaluation and validation studies were excluded from trend analysis because PubMed did not consistently index these publication types prior to 2001.

**Table 2:** Annual growth in total U.S., U.S. NIH-funded, and relative NIH-funded cardiovascular research articles, 1996–2006.

Methodology	All articles			NIH-funded articles			Relative proportion (NIH-funded/All)		
	Annual growth rate		*P*-value	Annual growth rate		*P*-value	Annual growth rate		*P*-value
	Articles	% of 1996		Articles	% of 1996		Δ ratio	% of 1996	
Case reports	31.7	12.9%	<0.001	0.31	5.2%	0.187	0.000	0.0%	0.557
Review articles	72.8	18.5%	<0.001	13.11	20.2%	<0.001	0.003	1.8%	0.076
Therapeutic comparisons	34.0	12.1%	0.001	10.64	15.6%	<0.001	0.005	2.1%	0.017
In vitro studies	−0.9	−1.4%	0.697	0.10	0.3%	0.954	0.010	1.9%	0.099
Meta-analyses	2.2	36.7%	<0.001	−0.12	-12.0%	0.224	-0.045	-27.0%	0.021
Multicenter studies	9.1	17.5%	<0.001	2.08	23.1%	<0.001	0.006	3.5%	0.092
Clinical trials, all types	15.4	12.3%	<0.001	4.44	17.1%	<0.001	0.007	3.4%	0.029
Controlled trials	2.8	31.1%	0.001	0.74	24.7%	<0.001	-0.002	−0.6%	0.842
Randomized-controlled trials	9.0	11.1%	<0.001	2.72	18.1%	<0.001	0.009	4.9%	0.007
Multicenter trials	4.4	11.3%	<0.001	0.72	9.0%	0.006	0.002	1.0%	0.597
Unspecified, general research	51.2	4.2%	0.003	32.71	8.1%	<0.001	0.009	2.7%	0.005
All journal articles	218.2	9.7%	<0.001	64.55	11.0%	<0.001	0.002	0.8%	0.345

All U.S. and NIH-funded U.S. growth rates are expressed in terms of articles per year, with relative growth expressed as a percentage of the 1996 level. The proportional growth in NIH-funded articles is expressed as the annual change in the ratio of NIH-funded to all articles, e.g. growth from 30% NIH-funded in 1996 to 41% NIH-funded in 2006 corresponds to a yearly growth of approximately 0.01, or 1 percent per year. Figures normalized to 1996 for this proportion are also depicted. Insufficient numbers of phase-type clinical trials were published to allow meaningful analysis of trend for these methodologies.

NIH-funded U.S. articles numbered 10,293 (28.1% of all U.S. articles). The NIH contributed heavily to general research (40.1%), and relatively less to clinical trials of any type (20.3%) and multicenter studies (17.6%). Support levels were particularly low for multicenter trials (12.2%), Phase II trials (11.1%), and Phase III and IV trials (both 0%), Table 1. NIH-funded articles grew by 64.6 articles per year (*P*<0.001). Significant growth was seen for NIH-funded general research articles, review articles, therapeutic comparisons, multicenter studies, and controlled, randomized-controlled, multicenter, and overall clinical trials, Table 2. No significant growth was detected for phase-type clinical trials.

**Figure 1:**
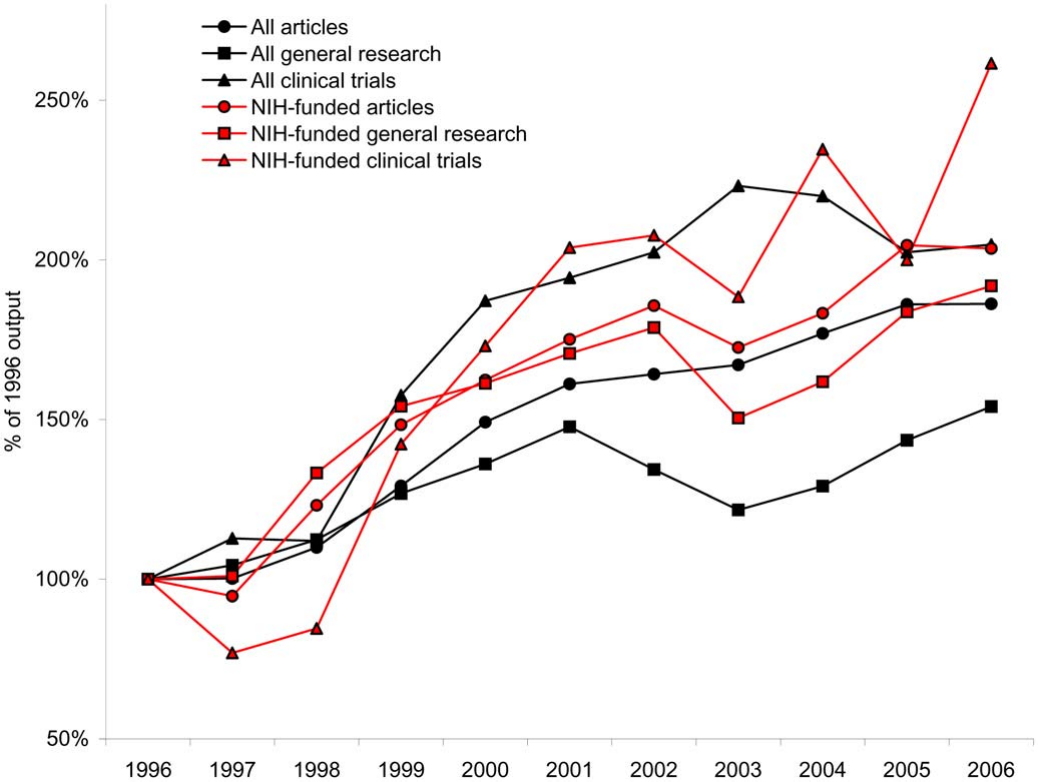
Trends in NIH-funded and overall U.S. cardiovascular disease articles, 1996–2006, selected methodologies. Data were normalized to 1996 levels. All article types depicted here had statistically significant growth except all U.S. general research articles. Furthermore the ratio of NIH-funded to overall articles increased significantly for general research articles and clinical trials, indicating a proportionally increasing role played by the NIH for these article types.

While numbers of both total and overall NIH-funded U.S. publications increased, there was no change in the relative proportion of NIH-funded articles. Statistically significant growth in this proportion was detected for a few specific methodologies, namely general research articles (0.87% per year, *P* = 0.005), therapeutic comparisons (0.52% per year, *P* = 0.017), randomized-controlled trials (0.92% per year, *P* = 0.007), and overall clinical trials (0.67% per year, *P* = 0.029). For these methodologies, the relative impact of the NIH expanded. Support for meta-analyses decreased (4.47% per year, *P* = 0.021), and all other methodologies showed no significant change, Table 2. Comparative growth in selected article methodologies is depicted in Figure 1.

**Figure 2:**
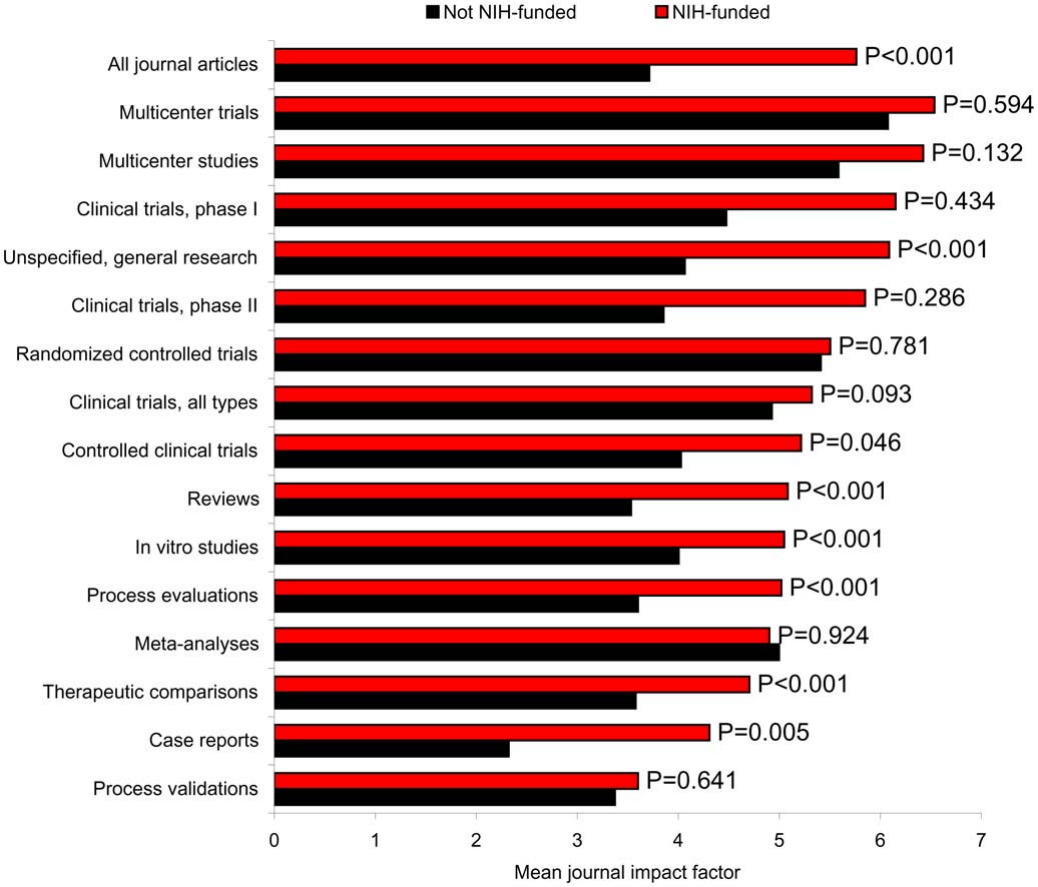
Mean journal impact factor of NIH-funded and non-NIH-funded U.S. cardiovascular disease articles, 1997 to 2006, by article methodology. *P*-values denote the difference between NIH-funded and non NIH-funded journal impact factors when means were compared with two-tailed t-tests. There was insufficient data for comparisons of Phase III and Phase IV clinical trials.

The mean journal impact factor of U.S. cardiovascular journal articles was 4.38, and was notably greater for rigorous methodologies such as multicenter trials (6.13), multicenter studies (5.74), randomized-controlled trials (5.43), and overall clinical trials (5.01). Case reports (2.36), process validations (3.44), and review articles (3.81) had the lowest impact factors. NIH-funded and non-NIH-funded articles had different mean journal impact factors in certain instances, as depicted in Figure 2. Impact factors were significantly greater for NIH-funded general research articles, case reports, review articles, in vitro studies, process evaluations, therapeutic comparisons, controlled clinical trials, and overall journal articles.

The leading NIH supporter of U.S cardiovascular publications was the National Heart, Lung and Blood Institute (NHLBI), which sponsored 8,898 publications, or 86.4% of all NIH-funded articles. Other major NIH supporters included the National Center for Research Resources (NCRR, 825 articles, 8.0%), the National Institute of Diabetes, & Digestive & Kidney Diseases (NIDDK, 680 articles, 6.6%), and the National Institute on Aging (NIA, 679 articles, 6.6%). The NHLBI demonstrated the greatest growth in publications, funding an additional 53.1 articles annually (*P*<0.001), followed by the NIA (10.5 articles/year, P<0.001) and NCRR (10.5 articles/year, *P*<0.001). A total of 12 NIH institutes exhibited significant growth in sponsored articles, Table 3. Of particular note was the National Institute of Biomedical Imaging and Bioengineering (NIBIB), the NIH institute most directly responsible for funding imaging and bioengineering research. Founded in December 2000, the 11-year trend analysis applied to other institutes is less appropriate for the NIBIB [22]. When a six-year analysis was performed on the six complete years of its existence, 2001 to 2006, the NIBIB exhibited an annual growth of 7.7 articles/year (P = 0.003), the fifth greatest growth rate among NIH institutes.

**Table 3:** Sponsored articles in cardiovascular disease research from individual NIH institutes, 1996–2006, with 11-year annualized growth rates.

NIH Institute	Abbrev.	Funded articles (%)	Growth (articles/yr)	*P*-value	95% CI
National Heart, Lung, and Blood Institute	NHLBI	8,898 (86.4%)	53.1	<0.001	(37.3–68.8)
National Center for Research Resources	NCRR	825 (8.0%)	10.5	<0.001	(8.1–12.9)
National Institute of Diabetes and Digestive and Kidney Diseases	NIDDK	680 (6.6%)	7.8	<0.001	(6.4–9.2)
National Institute on Aging	NIA	679 (6.6%)	10.5	<0.001	(7.8–13.3)
National Cancer Institute	NCI	459 (4.5%)	5.2	<0.001	(3.3–7.0)
National Institute of General Medical Sciences	NIGMS	416 (4.0%)	3.2	<0.001	(1.7–4.6)
National Institute of Arthritis and Musculoskeletal and Skin Diseases	NIAMS	255 (2.5%)	1.9	0.001	(1.0–2.8)
National Institute of Neurological Disorders and Stroke	NINDS	244 (2.4%)	0.9	0.151	(−0.4–2.2)
National Institute of Child Health and Human Development	NICHD	229 (2.2%)	2.3	0.004	(0.9–3.7)
National Institute of Allergy and Infectious Diseases	NIAID	183 (1.8%)	1.6	<0.001	(0.9–2.4)
National Institute of Biomedical Imaging and Bioengineering*	NIBIB	109 (1.1%)	7.7	0.003	(4.3–11.2)
National Eye Institute	NEI	91 (0.9%)	1.8	0.001	(0.9–2.6)
National Institute of Environmental Health Sciences	NIEHS	81 (0.8%)	0.7	0.062	(0.0–1.3)
National Institute on Drug Abuse	NIDA	49 (0.5%)	0.3	0.284	(−0.3–0.8)
National Institute of Mental Health	NIMH	47 (0.5%)	0.2	0.161	(−0.1–0.6)
National Institute on Alcohol Abuse and Alcoholism	NIAAA	44 (0.4%)	0.4	0.15	(−0.2–0.9)
John E. Fogarty International Center	FIC	25 (0.2%)	−0.4	0.024	(−0.7–−0.1)
National Institute of Nursing Research	NINR	21 (0.2%)	0.2	0.123	(−0.1–0.4)
National Institute on Deafness and Other Communication Disorders	NIDCD	19 (0.2%)	0.4	0.045	(0.0–0.9)
National Institute of Dental and Craniofacial Research	NIDCR	17 (0.2%)	0.2	0.306	(−0.2–0.5)

Individual articles may have received support from more than one institute, thus the sum of the percentiles from each institute exceeds 100%.

*The National Institute of Biomedical Imaging and Bioengineering (NIBIB) was created in December 2000, and thus no data were available for the first 5 years of the study. Trend analysis was performed on the 6 years for which data were available, 2001 to 2006.

## Discussion

The United States made a significant contribution to the world cardiovascular research literature, publishing slightly less than one third of all cardiovascular research articles over the study period. American investigators had a relative focus on expensive, high methodological strength trials, such as multicenter and phase-type trials. They accounted for more than one-third of the worldwide output of these trials, and in the case of Phase III trials, more than half. Perhaps as a result of the expertise and renown gained by this high profile research, U.S. investigators also published a disproportionately large and growing number of review articles. The bulk of worldwide clinical trials are of the non-multicenter variety, such as single center controlled or randomized-controlled clinical trials. The U.S. contributed less than a quarter of the world output of these trial types. Additionally, relatively few of the most inexpensive article type, case reports, originated from the U.S.

Despite the U.S. strength in large clinical trials, the NIH was less involved in sponsoring this type of research. While 28% of overall U.S. cardiovascular articles were NIH-funded, only 20.3% of clinical trials and 12.2% of multicenter trials received NIH sponsorship. The prohibitive cost of these trials may have been left to parties with the wealth and incentive to support them, namely industry. While this study did not directly investigate industrial sources of funding due to unstandardized reporting of industrial research support, it seems reasonable to assume that industry accounts for a large component of this funding gap, especially since in the general biomedical literature industrial support is twice that of the NIH [7]. Large clinical trials most directly and immediately impact clinical practice. Significant industry financing can improve and accelerate existing research and support ideas that might not otherwise be funded. On the other hand, conflict of interest and bias are important considerations when the sponsoring party has a financial interest in the research results [23], [24]. NIH support of clinical trials did exhibit significant growth, both in terms of absolute publications and relative proportion to total U.S. clinical trials. This growth coincided with the expansion of NIH and NHLBI budgets, the former of which more than doubled between 1998 and 2003 [5], [6]. Given the time lag between grant receipt and publication of final results for clinical trials, typically several years, it is possible that the NIH budget doubling was not fully expressed in the publications of this study period. If this is the case, then the period of budget stagnation from 2004 to 2008 was probably also not expressed. The full impact of NIH budget doubling, subsequent stagnation, and now ARRA stimulus boost remains to be seen. Since the overall trend is for clinical trials to be more dependent on NIH funding over time, the funding boost from the ARRA may have more beneficial impact than would otherwise be expected, especially if specific efforts are made to fund clinical trials with ARRA funds.

While NIH support of clinical trials is relatively limited, the converse is true for general research articles, 40.1% of which received NIH support. This proportion is far larger than any other article type. The basic science questions these articles typically examine may not yet be commercially viable. In this instance, the NIH may be filling a need for funding of discovery research that has no immediate commercial application, and is thus of lesser priority for corporate sponsors–a well-justified reason for government support [25]. Over the study period, overall general research articles grew significantly, but NIH-funded general research articles grew at a proportionally greater rate. Thus, the component of U.S. basic science and early clinical cardiovascular disease research that was directly funded by the NIH increased. These findings imply that fluctuation in NIH funding levels may have a disproportionately large impact on basic science research. Since basic science research precedes clinical research and ultimately clinical adoption, future clinical advances may be significantly compromised by the period of budget stagnation from 2003 to 2008, and significantly enhanced by the recent ARRA legislation.

The overall impact of NIH funding on publications grew steadily during the study period both overall and for many methodological types. The total number of NIH-funded articles more than doubled during the 11-year study period, increasing at 64.6 additional articles per year. The only methodologies that did not show growth in NIH-funded publications were meta-analyses, case reports, in vitro studies, and phase-type trials. During this same period, overall U.S. cardiovascular publication output grew by 218 articles per year, with significant growth seen for every methodological subtype except in vitro studies, the most rapidly growing of which was curiously review articles. Their growth may represent a growing need for articles to decipher and clarify an increasingly complex medical literature.

This growth in NIH-funded publications matched the growth in overall U.S. cardiovascular publications. The ratio of NIH-funded to overall U.S. articles, an indicator of relative growth, was essentially stable over the study period. Nominal but statistically significant increases in this proportion were detected for general research articles, randomized-controlled trials, and clinical trials of all types, with a sharp decrease seen for meta-analyses. Over this same period of time, the budget of the NHLBI grew from $1.35 billion in 1996 to $2.94 billion in 2005 [6]. This 217% increase in funding was matched by a doubling of NIH-funded articles over the same period. This growth was just enough to keep the NIH-funded component of all U.S. cardiovascular research stable at about 28%.

The impact factor of a publishing journal is predictive of the subsequent dissemination and likely scientific impact of its articles [21]. NIH-funded papers had greater journal impact factors than their non-NIH-funded cohorts, both overall and for numerous methodological subtypes, suggesting that these articles had a greater dissemination and scientific impact when compared with other similar articles. The reason for this difference is unknown. One possible explanation is that the NIH effect is directly causative, that is, the additional resources and prestige that come with NIH support improve the quality or perceived quality of the research, enabling publication in more prestigious journals. Another possibility is a correlative relationship. For instance, the intense scrutiny of the grant application process may select for more talented investigators who subsequently produce better research and publish in more prestigious journals on their merit alone. Alternatively, a combination of effects may be at play. While predictive of greater journal impact for other article types, NIH-funding did not predict a statistically significant difference in journal impact for any clinical trial type except controlled clinical trials. This finding again supports the notion that the NIH is not unduly influential in clinical trials, where NIH sponsorship is relatively low. In some instances, such as with Phase I and II trials, the small number of NIH-funded studies made detection of all but the largest differences difficult.

The NHLBI was by far the dominant NIH institute providing funding for cardiovascular research. It was involved in more than 6 out of 7 NIH-sponsored cardiovascular publications and exhibited the greatest rate of support growth. The NIA and NIDDK also exhibited strong historical support and growth, an unsurprising result given the strong relationships between diabetes, aging, and heart disease. Of interest is the relatively rapid growth in support from the NIBIB, the institute most directly responsible for imaging and bioengineering research. Founded in December, 2000, and the youngest of the NIH institutes, the NIBIB is strongly associated with clinical research in radiology [22]. The fact that its growth in cardiovascular disease research is so prominent, suggests that cardiac imaging may be an important funding priority. Cardiovascular disease investigators working with imaging should consider the NIBIB as a potential source of grant support.

### Study Limitations

There are several limitations of this study that are important to consider. First, data analyzed were limited to publications indexed in the National Library of Medicine's PubMed database. PubMed indexes about 5,200 journals published in 80 countries, and contains more than 18.8 million citations [14], [16]. While very comprehensive, PubMed is not perfect, and any bias in journal inclusion or accuracy of data field indexing could impact the study results. Generally however, PubMed is widely considered to be an accurate and reliable resource. Another consideration is the use of the first author's affiliation to assign the country and department of origin. Articles pertaining to cardiovascular disease in which the first author is not from a cardiovascular or related department would not have been counted. Furthermore, articles where the first author is from a cardiovascular department but is writing about an unrelated topic would be erroneously included. On the other hand, giving equal credit to all authors in multiauthor papers runs the risk of overestimating the contribution of non-first authors. Several studies have shown that first authors make the most substantial contributions to the published work, and are the authors most deserving of credit [26], [27]. Journal impact factor, while indicative of a journal's prestige and likely scientific impact of its articles in aggregate, is not necessarily indicative of an individual article's scientific impact. This should be kept in mind when interpreting JIF results. Finally, the paper makes no attempt to account for variation in the competitiveness of NIH funding in different study years.

### Conclusions

In summary, this study provides a comprehensive overview of the impact of NIH funding on published U.S. cardiovascular disease research. By including 36,684 U.S. cardiovascular disease articles published during the 11-year study period, the data set was very comprehensive. The U.S. accounted for about one third of worldwide publications on cardiovascular disease, with a relative emphasis on large clinical trials and review articles. The NIH funded 28% of U.S. articles, with an emphasis on basic science research. Most large U.S. clinical trials received alternative funding, likely from industrial sources. NIH-funded articles of several methodologies were more likely to be published in high impact journals. Both overall U.S. cardiovascular publications and NIH-funded publications increased, but at roughly equivalent rates such that the ratio between the two was stable. The NHLBI was by far the dominant institute funding cardiovascular research, but growth from the NIBIB was unexpectedly strong, suggesting a growing federal interest in cardiac imaging.
